# The relation of the frequency and functional molecules expression on plasmacytoid dendritic cells to postpartum hepatitis in women with HBeAg-positive chronic hepatitis B virus infection

**DOI:** 10.3389/fimmu.2022.1062123

**Published:** 2022-11-09

**Authors:** Fuchuan Wang, Meiying Song, Yuhong Hu, Liu Yang, Xiaoyue Bi, Yanjie Lin, Tingting Jiang, Wen Deng, Shiyu Wang, Fangfang Sun, Zhan Zeng, Yao Lu, Ge Shen, Ruyu Liu, Min Chang, Shuling Wu, Yuanjiao Gao, Hongxiao Hao, Mengjiao Xu, Xiaoxue Chen, Leiping Hu, Gang Wan, Lu Zhang, Minghui Li, Yao Xie

**Affiliations:** ^1^ Department of Gynecology and Obstetrics, Beijing Ditan Hospital, Capital Medical University, Beijing, China; ^2^ Department of Gynecology and Obstetrics, Fu Xing Hospital, Capital Medical University, Beijing, China; ^3^ Department of Hepatology Division 2, Beijing Ditan Hospital, Capital Medical University, Beijing, China; ^4^ Department of Hepatology Division 2, Peking University Ditan Teaching Hospital, Beijing, China; ^5^ Department of Medical Records, Beijing Ditan Hospital, Capital Medical University, Beijing, China

**Keywords:** plasmacytoid dendritic cells, chronic hepatitis B, antiviral treatment, postpartum, immunity

## Abstract

**Objective:**

To explore the correlation between postpartum hepatitis and changes of plasmacytoid dendritic cells’ (pDC) function and frequency in hepatitis B e antigen (HBeAg)-positive pregnant women with chronic hepatitis B virus (HBV) infection.

**Methods:**

Pregnant women with chronic HBV infection receiving antiviral treatment (treated group) or not receiving antiviral treatment (untreated group) were enrolled and demographic information was collected before delivery. Clinical biochemical, virological serology, pDC frequency and functional molecular expression were tested before delivery and at 6, 12, 24 weeks after delivery.

**Results:**

90 eligible pregnant women were enrolled, 36 in the untreated group and 54 in the treated group. 36 patients developed postpartum hepatitis, including 17 (17/36, 47.2%) in the untreated group and 19 (19/54, 35.2%) in the treated group (χ2 = 1.304 p=0.253), and 22 cases of hepatitis occurred at 6 weeks postpartum, 12 at 12 weeks postpartum, and 2 at 24 weeks postpartum. The alanine transaminase (ALT) levels at any time postpartum were significantly higher than that of the antepartum, especially at 6 weeks and 12 weeks postpartum. However, the frequencies of pDCs, CD83^+^ pDCs and CD86^+^ pDCs antepartum had no significant difference from any time postpartum. The frequencies of CD83^+^ pDCs, CD86^+^ pDCs in the treated group antepartum were significantly higher than those in the untreated group [12.70 (9.46, 15.08) vs. 10.20 (7.96, 11.85), p=0.007; 22.05 (19.28, 33.03) vs. 18.05 (14.33, 22.95), p=0.011], and the same at 12 weeks postpartum [12.80 (10.50, 15.50) vs. 9.38 (7.73, 12.60), p=0.017; 22.50 (16.80, 31.20) vs. 16.50 (12.65, 20.80), p=0.001]. The frequency of CD86^+^ pDCs in the treated group was significantly higher than that in the untreated group at 24 weeks postpartum [22.10 (16.70, 30.00) vs. 17.10 (13.70, 20.05), p=0.006].

**Conclusions:**

Postpartum hepatitis in HBV infected women mainly occurs at 6-12 weeks postpartum. Antiviral treatment during pregnancy can significantly increase the frequencies of CD83^+^ pDCs and CD86^+^ pDCs in pregnant women with chronic HBV infection.

## Introduction

Mother-to-child transmission (MTCT) of HBV is the main cause of chronic HBV infection in China. The rate of HBV MTCT maintained 7%-11% in newborns of HBeAg-positive mothers despite combined active-passive immunization with a hepatitis B vaccine and hepatitis B immunoglobulin (HBIG) shortly after birth. HBeAg positive and high HBV DNA level (HBV DNA > 10^5^ IU/ml) in pregnant women are the main cause of HBV MTCT (1, 2). Antiviral treatment during the third trimester can significantly reduce the rate of MTCT in pregnant women with high HBV DNA level (3, 4).

pDCs can secrete a large amount of cytokine interferon (IFN) with pleiotropic effect during viral infection, and they have a strong ability to activate the initial immune response and activate natural killer (NK) cells and T lymphocytes to further activate and regulate the antiviral immune response (5). They play a vital role in the control and prognosis of HBV infection. Studies have shown that hepatitis B surface antigen (HBsAg) and HBeAg can inhibit the frequency and function of pDCs in chronic HBV infected patients, leading to the impairment of antigen presenting function of pDCs (6, 7).

The elevation of postpartum ALT is a common phenomenon. It is believed that due to the decrease of steroid hormone levels after delivery, the immunosuppressive state during pregnancy is rapidly relieved, triggering the immune response to HBV and mediating liver tissue injury and hepatocyte necrosis in patients with chronic hepatitis B (CHB). Hepatocyte necrosis leads to elevation of ALT and indicates that these women have entered the immune clearance period or the reactivation period (8), which is a good time to treat hepatitis (7, 9).

However, there are few studies on the changes of postpartum immune cell function of HBeAg-positive pregnant women with chronic HBV infection and its association with postpartum hepatitis, and few reports about the effects of antiviral treatment during pregnancy on postpartum immune cell function and postpartum hepatitis. Therefore, we designed a prospective case-cohort study and recruited HBeAg-positive chronic HBV infected pregnant women after informed consent. We measured the frequency of pDCs and the expression of functional molecules in peripheral blood antepartum and at 6, 12, 24 weeks postpartum to explore the frequency and functional changes of pDCs after delivery.

## Materials and methods

### Research objects

Pregnant women with chronic HBV infection were enrolled in the study from January 2016 to January 2018. Inclusion criteria: 1. Between the ages of 20 and 40; 2. HBsAg and HBeAg positive over 6 months, and serum HBV DNA > 2.0×10^5^ IU/ml; 3. Liver function remained normal; 4. With or without antiviral treatment at 24-28 weeks of gestation as they wish. Exclusion criteria: 1. History of amniocentesis during pregnancy; 2. History of hereditary diseases in one or both families of the couple; 3. Fetal abnormalities detected before enrollment by imaging or other prenatal tests, including genetic testing; 4. Coinfection with hepatitis C virus, hepatitis D virus, human immunodeficiency virus, syphilis, toxoplasmosis, rubella, or cytomegalovirus; 5. History of two or more spontaneous abortions; 6. Previous delivery of a child with deformity; 7. Twin or multiple pregnancies; 8. The mother had a history of cancer or autoimmune diseases.

Pregnant women were classified into treated group (with antiviral treatment at 24-28 weeks of gestation) and untreated group (without antiviral treatment during pregnancy). Antiviral therapy was stopped immediately after delivery and all women were followed up at 6, 12 and 24 weeks postpartum.

All subjects signed an informed consent. The study was approved by the ethics committee of Beijing Ditan Hospital, Capital Medical University (JDL-2017-004-01), and registered at clinicaltrials.gov (Clinical registration No: NCT03214302).

### Blood specimen collection

2 ml of peripheral venous blood of all pregnant women was collected by EDTA anticoagulant violet tube at the time of antepartum (baseline) and 6, 12, 24 weeks postpartum, cells were collected by BD FACS Calibur within 4 hours of blood collection.

### Virological indexes and clinical biochemical indicators

Virological indexes (HBeAg, HBsAg and HBV DNA) and ALT, glutamic oxaloacetic transaminase (AST), total bilirubin (TBIL), albumin (ALB) were tested by the laboratory of Beijing Ditan Hospital, Capital Medical University.

### pDC functional detection

pDCs were detected with Lineage1-FITC, HLA-DR-PerCP, Anti-CD123-APC, Anti-CD83-PE, Anti-CD86-PE antibodies, and CD83MFI and CD86MFI data were analyzed by FlowJo7.6 software.

### Statistical analysis

Data were analyzed by SPSS 19.0 software. All data were presented as mean ± standard deviation (mean ± SD) or median, interquartile interval [median (Q1, Q3)], box-and-whisker plots or percentage. Student t-test was used for normal distribution data and Mann-Whitney test was used for skewness distribution data between groups. P < 0.05 was considered statistically significant.

## Results

### Baseline characteristics

90 pregnant women were enrolled, including 54 cases with antiviral treatment (treated group) and 36 cases without antiviral treatment (untreated group). There was no difference between the two groups in age, gestational weeks, pregnancy complications, neonatal weight, length, Apgar score at 5 minutes and neonatal malformations (P>0.05). The number of gravidity and parity in the untreated group were significantly higher than those in the treated group (p=0.015 and 0.005, respectively). The level of TBIL was 7.05±2.18umol/l in the treated group and 8.18±2.73umol/l in the untreated group (P=0.033), but TBIL in both groups was within the normal range. The levels of HBsAg, HBeAg and HBV DNA antepartum in the treated group were significantly lower than those in the untreated group (P<0.05), which was related to antiviral therapy. The basic information is shown in **Table 1**.

**Table 1:** Basic characteristics of the subjects.

Values	All patients (n=90)	Treated group (n=54)	Untreated group (n=36)	t/χ2	P value
Mothers					
Ages (year)	29.79±3.86	30.20±4.20	29.17±3.24	-1.254	0.213
No. of gravidity (times)	2.17±1.07	1.94±1.02	2.50±1.08	2.474	0.015*
No. of parity (times)	1.50±0.55	1.37±0.49	1.69±0.58	2.870	0.005*
Gestational weeks (weeks)	39.24±1.03	39.41±1.00	39.00±1.04	-1.862	0.066
Vaginal delivery(%)	51(56.67%)	34(62.96%)	17(47.22%)	2.179	0.140
precipitate labour (%)	4(4.44%)	4(7.41%)	0(0%)	Fieher	0.147
Postpartum hemorrhage (%)	5(5.56%)	3(5.56%)	2(5.56%)	Fisher	1.000
Hypertension disorder of pregnancy (%)	1(1.11%)	1(1.85%)	0(0%)	Fisher	1.000
Diabetes (%)	24(26.67%)	15(27.78%)	9(25%)	0.085	0.770
Hypothyroidism (%)	3(3.33%)	2(3.7%)	1(2.78%)	Fisher	1.000
Intrahepatic cholestasis of pregnancy (%)	1(1.11%)	1(1.85%)	0(0%)	Fisher	1.000
Anemia (%)	3(3.33%)	2(3.7%)	1(2.78%)	Fisher	1.000
ALT level (U/L)	22.51±29.05	20.46±8.84	25.60±44.84	0.679	0.502
AST level (U/L)	23.58±18.12	22.95±5.91	24.53±27.95	0.333	0.741
TBIL level (umol/L)	7.50±2.46	7.05±2.18	8.18±2.73	2.161	0.033*
ALB level (g/L)	36.32±2.17	36.26±2.26	36.40±2.07	0.293	0.770
HBsAg level (log_10_ IU/ml)	4.40±0.41	4.29±0.42	4.56±0.35	3.127	0.002*
HBeAg level (S/CO)	1360.43±442.35	1207.54±465.36	1589.77±281.92	4.847	0.000*
HBV DNA level (log_10_ IU/ml)	5.79±2.02	4.28±0.96	8.06±0.33	26.577	0.000*
Infants					
Male (%)	52(50.00%)	27(50.00%)	19(52.77%)	0.067	0.796
Length (cm, mean ± SD)	50.09±0.83	50.11±1.02	50.06±0.41	-0.310	0.758
Weight (kg, mean ± SD)	3338.33±395.93	3297.04±435.00	3400.28±324.79	1.215	0.228
Apgar score at 5 min (mean ± SD)	9.97±0.32	10.00±0.00	9.92±0.50	-1.000	0.324
Neonatal malformations (%)	3(3.33%)	1(1.85%)	2(5.55%)	Fisher	0.561

*P < 0.05, was considered statistically significant.

### Changes of pDC and ALT after delivery

In this study we use ALT≥ 2 times the upper limit of normal (40 U/L) as the diagnostic criterion for the occurrence of hepatitis according to the Asian-Pacific clinical practice guidelines on the management of hepatitis B (10). The ALT levels of all pregnant women enrolled were significantly higher at 6, 12 and 24 weeks postpartum than those of antepartum (P<0.05), postpartum hepatitis occurred in 36 cases, 22 cases occurred at 6 weeks postpartum, 12 at 12 weeks postpartum, 2 at 24 weeks postpartum. 17 cases (17/36) of abnormal ALT occurred in the untreated group and 19 (19/54) occurred in the treated group, with no significant difference (χ2 = 1.304, p=0.253). The frequencies of antepartum pDCs, CD83^+^ pDCs and CD86^+^ pDCs in all pregnant women enrolled were not significantly different from those at 6, 12 and 24 weeks postpartum (P > 0.05) (**Table 2**).

**Table 2:** ALT and pDC levels antepartum and postpartum of all patients.

Values	0 week	6 week	12 week	24 week	Z or T value/P value
ALT (U/L)	17.15 (13.58, 22.63)	45.35 (28.63, 85.00)	55.30 (32.60, 96.60)	29.00 (23.18, 42.93)	a1=-9.362/0.000* a2=-9.590/0.000* a3=-6.968/0.000*
pDC/PBMC (%)	0.20 (0.14, 0.26)	0.20 (0.14, 0.25)	0.18 (0.13, 0.25)	0.21 (0.16, 0.29)	a1=-0.086/0.932 a2=-0.781/0.435 a3=-1.510/0.131
CD83+pDC (%)	11.20 (8.48, 14.35)	11.70 (9.18, 14.25)	12.25 (9.34, 14.45)	10.25 (8.50, 12.18)	a1=-0.452/0.651 a2=-1.033/0.302 a3=-1.753/0.080
CD83MFI (%)	18.30 (14.7, 21.6)	17.90 (15.35, 22.40)	18.00 (15.88, 23.25)	18.35 (15.50, 21.40)	a1=-0.708/0.479 a2=-0.753/0.451 a3=-0.354/0.723
CD86+pDC (%)	20.50 (16.78, 28.5)	19.80 (15.45, 26.95)	19.50 (15.48, 27.73)	19.55 (15.78, 26.98)	a1=-0.504/0.614 a2=-0.796/0.426 a3=-0.908/0.364
CD86MFI (%)	87.00 (72.15, 104.25)	88.20 (75.35, 106.00)	89.00 (71.70, 115.00)	99.10 (78.78, 109.75)	a1=-0.322/0.747 a2=-0.798/0.425 a3=-1.773/0.076

1. 0 week=antepartum 6 week=6 weeks postpartum 12 week=12 weeks postpartum 24 week=24 weeks postpartum.

2. a1: 0 week vs. 6 week a2: 0 week vs. 12 week a3: 0 week vs. 24 week.

3. * P < 0.05, was considered statistically significant.

4. pDCs frequency and function include pDC%, CD83^+^pDC%, CD83MFI and CD86^+^ pDC%, CD86MFI.

5. The expression of CD83 and CD86 molecules on the surface of pDC cells is also the mean fluorescence intensity (MFI).

### Frequency and expression of functional molecules of pDC cells and ALT in HBV infected pregnant women postpartum

The ALT levels were significantly higher at 6, 12, and 24 weeks postpartum than those of the antepartum in both treated and untreated group (p<0.001). In the treated group, the frequency of CD83^+^ pDCs antepartum was significantly higher than that of the 24 weeks postpartum [12.70(9.46, 15.08) vs. 10.40(9.39, 12.10), p=0.026]. In the untreated group, the frequency of CD83^+^ pDCs antepartum was significantly lower than that of the 6 weeks postpartum [10.20 (7.96, 11.85) vs. 11.70 (9.12, 14.23), p=0.040] (**Table 3**).

**Table 3:** The changes of postpartum ALT and pDC levels in Treated and Untreated group.

Values	Treated group (n=54)					Untreated group (n=36)				
	0 week	6 week	12 week	24 week	Z or T/P value	0 week	6 week	12 week	24 week	Z or T/P value
ALT (U/L)	17.95 (14.85, 24.03)	40.25 (28.18, 62.68)	49.35 (31.28, 78.80)	27.85 (23.63, 35.90)	a1=-6.925/0.000* a2=-7.549/0.000* a3=-4.889/0.000*	15.10 (12.00, 21.68)	53.40 (30.73, 157.45)	68.30 (40.55, 146.80)	33.75 (23.00, 58.00)	a1=-6.163/0.000* a2=-5.810/0.000* a3=-4.848/0.000*
pDC/PBMC (%)	0.20 (0.15, 0.26)	0.20 (0.16, 0.25)	0.19 (0.13, 0.26)	0.21 (0.17, 0.30)	a1=-0.347/0.728 a2=-0.164/0.870 a3=-1.328/0.184	0.19 (0.13, 0.26)	0.18 (0.12, 0.25)	0.18 (0.11, 0.23)	0.22 (0.12, 0.28)	a1=-0.628/0.530 a2=-1.250/0.211 a3=-0.654/0.513
CD83^+^ pDC (%)	12.70 (9.46, 15.08)	11.70 (9.27, 14.30)	12.80 (10.50, 15.50)	10.40 (9.39, 12.10)	a1=-1.047/0.295 a2=-0.689/0.491 a3=-2.230/0.026*	10.20 (7.96, 11.85)	11.70 (9.12, 14.23)	9.38 (7.73, 12.60)	10.10 (5.59, 12.30)	a1=-2.053/0.040* a2=-0.128/0.898 a3=-0.422/0.673
CD83MFI (%)	18.70 (15.23, 22.6)	19.30 (16.45, 23.70)	19.80 (16.70, 24.80)	19.80 (15.80, 24.80)	a1=-0.773/0.440 a2=-1.223/0.221 a3=-0.735/0.462	16.75 (14.40, 19.05)	16.65 (14.20, 20.50)	16.50 (13.55, 18.25)	16.70 (14.10, 19.15)	a1=-0.081/0.936 a2=-0.599/0.549 a3=-0.314/0.753
CD86^+^ pDC (%)	22.05 (19.28, 33.03)	20.80 (16.65, 29.05)	22.50 (16.80, 31.20)	22.10 (16.70, 30.00)	a1=-1.218/0.223 a2=-0.592/0.554 a3=-0.760/0.447	18.05 (14.33, 22.95)	18.25 (14.48, 25.05)	16.50 (12.65, 20.80)	17.10 (13.70, 20.05)	a1=-0.644/0.519 a2=-0.932/0.351 a3=-0.596/0.551
CD86MFI (%)	90.60 (72.83, 114.25)	89.00 (80.25, 106.00)	94.70 (77.00, 118.00)	104.00 (82.00, 113.00)	a1=-0.088/0.932 a2=-1.047/0.295 a3=-1.273/0.203	83.95 (69.13, 93.70)	85.85 (68.68, 107.25)	78.40 (67.30, 103.50)	89.80 (78.05, 104.50)	a1=-0.299/0.765 a2=-0.539/0.590 a3=-1.332/0.183

1. 0 week=antepartum 6 week=6 weeks postpartum 12 week=12 weeks postpartum 24 week=24 weeks postpartum.

2. a1: 0 week vs. 6 week a2: 0 week vs. 12 week a3: 0 week vs. 24 week, they were comparsion within groups.

3. * P < 0.05, was considered statistically significant.

4. pDCs frequency and function include pDC%, CD83^+^ pDC%, CD83MFI and CD86^+^ pDC%, CD86MFI.

### Effect of antiviral therapy on the frequency and the expression of functional molecules of pDCs and ALT

The frequencies of CD83^+^ pDCs and CD86^+^ pDCs in the treated group were significantly higher than those in the untreated group before delivery [12.70 (9.46, 15.08) vs. 10.20 (7.96, 11.85), p=0.007; 22.05 (19.28, 33.03) vs. 18.05 (14.33, 22.95), p=0.011]. The frequencies of CD83^+^ pDCs and CD86^+^ pDCs in the treated group at 12 weeks postpartum were significantly higher than those in the untreated group [12.80 (10.50, 15.50) vs. 9.38 (7.73, 12.60), p=0.017; 22.50 (16.80, 31.20) vs. 16.50 (12.65, 20.80), p=0.001]. The frequency of CD86^+^ pDCs in the treated group at 24 weeks postpartum was significantly higher than that in the untreated group [22.10 (16.70, 30.00) vs. 17.10 (13.70, 20.05), p=0.006]. It is suggested that antiviral treatment in the third trimester of pregnancy can significantly increase the frequencies of CD83^+^ pDCs and CD86^+^ pDCs in peripheral blood antepartum and postpartum (**Table 4**).

**Table 4:** Comparison of ALT and pDC levels at different time points between two groups.

Values	Treated group (n=54)				Untreated group (n=36)				T value/P value
	0 week	6 week	12 week	24 week	0 week	6 week	12 week	24 week	
ALT (U/L)	17.95 (14.85, 24.03)	40.25 (28.18, 62.68)	49.35 (31.28, 78.80)	27.85 (23.63, 35.90)	15.10 (12.00, 21.68)	53.40 (30.73, 157.45)	68.30 (40.55, 146.80)	33.75 (23.00, 58.00)	a1 = 0.679/0.502 a2 = 1.717/0.095 a3 = 1.724/0.097 a4 = 1.547/0.133
pDC/PBMC (%)	0.20 (0.15, 0.26)	0.20 (0.16, 0.25)	0.19 (0.13, 0.26)	0.21 (0.17, 0.30)	0.19 (0.13, 0.26)	0.18 (0.12, 0.25)	0.18 (0.11, 0.23)	0.22 (0.12, 0.28)	a1=-0.138/0.891 a2=-0.405/0.686 a3=-1.425/0.159 a4=-0.686/0.496
CD83^+^ pDC (%)	12.70 (9.46, 15.08)	11.70 (9.27, 14.30)	12.80 (10.50, 15.50)	10.40 (9.39, 12.10)	10.20 (7.96, 11.85)	11.70 (9.12, 14.23)	9.38 (7.73, 12.60)	10.10 (5.59, 12.30)	a1=-2.786/0.007* a2 = 0.731/0.467 a3=-2.446/0.017* a4=-1.075/0.291
CD83MFI (%)	18.70 (15.23, 22.6)	19.30 (16.45, 23.70)	19.80 (16.70, 24.80)	19.80 (15.80, 24.80)	16.75 (14.40, 19.05)	16.65 (14.20, 20.50)	16.50 (13.55, 18.25)	16.70 (14.10, 19.15)	a1=-1.260/0.211 a2=-0.189/0.851 a3=-2.811/0.007* a4=-1.966/0.054
CD86^+^ pDC (%)	22.05 (19.28, 33.03)	20.80 (16.65, 29.05)	22.50 (16.80, 31.20)	22.10 (16.70, 30.00)	18.05 (14.33, 22.95)	18.25 (14.48, 25.05)	16.50 (12.65, 20.80)	17.10 (13.70, 20.05)	a1=-2.594/0.011* a2=-1.410/0.162 a3=-3.393/0.001* a4=-2.867/0.006*
CD86MFI (%)	90.60 (72.83, 114.25)	89.00 (80.25, 106.00)	94.70 (77.00, 118.00)	104.00 (82.00, 113.00)	83.95 (69.13, 93.70)	85.85 (68.68, 107.25)	78.40 (67.30, 103.50)	89.80 (78.05, 104.50)	a1=-1.378/0.172 a2=-0.565/0.574 a3=-2.062/0.044* a4=-1.527/0.133

1. 0 week=antepartum 6 week=6 weeks postpartum 12 week=12 weeks postpartum 24 week=24 weeks postpartum.

2. a1: 0 week vs. 0week a2: 6 week vs. 6 week a3: 12 week vs. 12 week a4: 24 week vs. 24 week. they were comparsion between groups.

3. * P < 0.05, was considered statistically significant.

4. pDCs frequency and function include pDC%, CD83^+^ pDC%, CD83MFI and CD86^+^ pDC%, CD86MFI.

## Discussion

Dendritic cells (DCs) can be divided into pDCs and myeloid dendritic cells (mDCs) according to the source of precursor cells. According to specific functions, DCs can be divided into immature DCs and mature DCs, most of which are immature DCs under normal circumstances and have a strong ability to capture antigens (11, 12). However, the surface of immature DCs lacks some costimulatory molecules, which cannot provide necessary costimulatory signals for T cell activation, making T cells unable to be fully activated and in a state of loss of function, meanwhile, immature DCs also induce antigen specific regulatory T cells, block the functional response of effector T cells, and also can induce immune tolerance (12–14). Mature DCs stimulate T lymphocytes by expressing major histocompatibility complexes and costimulatory molecules, and secreting cytokines such as IFN, thus initiating the cellular immune response (14, 15). At present, research suggests that CD86 is a marker of DC activation, and CD83 is a specific marker of DC maturation. DC activation and maturation increase the expression of cell surface molecules CD86 and CD83, otherwise inhibit the differentiation and maturation of DCs, and reduce the expression of cell surface molecules CD86 and CD83 (16–19).

Recently, the function of pDCs has attracted extensive attention in the field of chronic HBV infection. As the only innate immune cells with antigen presenting ability, which can activate non-sensitized initial T cells, pDC is the main effector cell of human antiviral immunity and plays a double-acting role in antiviral treatment, which can mediate both immune response and immune tolerance (12, 14, 16). Mature pDCs can secrete a large amount of cytokines with pleiotropic effects to directly inhibit viral replication during viral infection, and can also activate mDCs, T cells, NK cells and B lymphocytes to form protective immune response, becoming a bridge connecting natural immunity and acquired immunity, with strong ability to activate initial immune response. Immature pDCs have a strong ability to capture antigen, but the ability to present antigen is weak, making T cells cannot be fully activated, and have a strong ability to induce regulatory T cells, which mediates the induction and maintenance of immune tolerance (14–18).

Studies have suggested that HBsAg and HBeAg in patients with chronic HBV infection can inhibit the function of DCs, which leads to a decrease in the frequency and function of pDCs in patients with chronic HBV infection (20, 21). Our previous study on the immune function of HBeAg positive chronic hepatitis B patients found that the frequency of pDCs was related to the incidence of CHB, the lower the frequency of pDCs in CHB patients, the more likely they were to develop hepatitis. However, we did not distinguish immature pDCs and mature pDCs frequency changes on the impact of hepatitis in this study (7, 22). Our previous retrospective study and this prospective study both suggest that postpartum liver dysfunction is common in women with chronic HBV infection, we also found that the abnormal liver function of HBV infected women mainly occurred in 6-12 weeks postpartum (23, 24), which indicated the importance of preventing postpartum hepatitis. We further studied the effect of antiviral therapy in the third trimester on postpartum hepatitis, and found that with the reduction of HBV DNA level due to the effective antiviral therapy during pregnancy, the inhibition of the virus on the pDCs function was alleviated. Based on this result, we also studied the effect of antiviral treatment in the third trimester of HBeAg-positive chronic HBV infected women on the frequency and function of postpartum peripheral blood pDCs and ALT level. Our study found that the ALT level of the pregnant women receiving antiviral treatment in the third trimester has a downward trend at 6-12 weeks postpartum, but there is no statistical significance between the two groups, which may be related to the limited number of patients in our study. Our study also found that there was no significant change in the frequency of pDCs after antiviral treatment during pregnancy, but it could significantly increase the frequencies of CD83^+^ pDCs and CD86^+^ pDCs in pregnant women with chronic HBV infection before delivery and 12 weeks postpartum. This might indicate that antiviral therapy in late pregnancy increases the proportion of mature pDCs, the immune activity and antiviral function of pDCs, which may improve the success rate of postpartum hepatitis treatment. However, this study had a small sample, and only involved the changes of pDCs in peripheral blood, without discussing the changes of the immune function in the liver, therefore, if we want to elucidate the above causal relationship, we need to further enlarge the sample and investigate the changes of the immune function in liver, as well as in peripheral blood, which would shed new light on the prevention and treatment of postpartum hepatitis in HBeAg positive chronic HBV infected pregnant women.

## Data availability statement

The raw data supporting the conclusions of this article will be made available by the authors, without undue reservation.

## Ethics statement

The studies involving human participants were reviewed and approved by The ethics committee of Beijing Ditan Hospital, Capital Medical University. The patients/participants provided their written informed consent to participate in this study.

## Author contributions

FW, GW, LZ, ML, and YX contributed to study concept and design. MS, YH, YLi, XB, YLu, TJ, WD, SyW, FS, ZZ, GS, RL, MC, SlW, YG, HH, MX, XC, and LH performed the data collection. FW, GW, LZ, ML, and YX conducted data analysis. FW, MS, YH, LY, and XB wrote the first draft. FW edited the English version. GW, LZ, ML, and YX approved the submitted version after modification. All authors contributed to the article and approved the submitted version.

## Funding

Project supported by Beijing Science and Technology Commission (Z211100002921059). National Science and Technology Major Project of China (2017ZX10201201-001-006, 2017ZX10201201-002-006, 2018ZX10715-005-003-005). The Capital Health Research and Development of Special (2022–1–2172). The Digestive Medical Coordinated Development Center of Beijing Hospitals Authority (XXZ0302 and XXT28). Beijing Hospitals Authority Clinical Medicine Development of Special Funding Support (XMLX 202127). High-level Public Health Technical Personnel Training Program of Beijing Municipal Health Commission (2022–3–050).

## Conflict of interest

The authors declare that the research was conducted in the absence of any commercial or financial relationships that could be construed as a potential conflict of interest.

The reviewer XW declared a shared affiliation with the authors to the handling editor at the time of review.

## Publisher’s note

All claims expressed in this article are solely those of the authors and do not necessarily represent those of their affiliated organizations, or those of the publisher, the editors and the reviewers. Any product that may be evaluated in this article, or claim that may be made by its manufacturer, is not guaranteed or endorsed by the publisher.
